# What does quality of life mean to older adults? A thematic synthesis

**DOI:** 10.1371/journal.pone.0213263

**Published:** 2019-03-08

**Authors:** Karen M. van Leeuwen, Miriam S. van Loon, Fenna A. van Nes, Judith E. Bosmans, Henrica C. W. de Vet, Johannes C. F. Ket, Guy A. M. Widdershoven, Raymond W. J. G. Ostelo

**Affiliations:** 1 Department of Health Sciences, Faculty of Science, Vrije Universiteit Amsterdam, Amsterdam Public Health Research Institute, Amsterdam, The Netherlands; 2 Department of Medical Humanities, Amsterdam UMC, Amsterdam Public Health Research Institute, Amsterdam, The Netherlands; 3 ACHIEVE Centre of Applied Research, Faculty of Health, Amsterdam University of Applied Sciences, Amsterdam, The Netherlands; 4 Department of Epidemiology and Biostatistics, Amsterdam UMC, Amsterdam Public Health Research Institute, Amsterdam, The Netherlands; 5 Medical Library, Vrije Universiteit, Amsterdam, The Netherlands; Nathan S Kline Institute, UNITED STATES

## Abstract

**Background:**

Consideration of older adults’ quality of life (QoL) is becoming increasingly important in the evaluation, quality improvement and allocation of health and social care services. While numerous definitions and theories of QoL have been proposed, an overall synthesis of the perspective of older adults themselves is lacking.

**Methods:**

Qualitative studies were identified in PubMed, Ebsco/Psycinfo and Ebsco/CINAHL, through a search on 28 November 2018. Articles needed to meet all of the following criteria: (i) focus on perceptions of QoL, (ii) older adults living at home as main participants, (iii) use of qualitative methodology, (iv) conducted in a Western country and (v) published in English (vi) not focused on specific patient groups. A thematic synthesis was conducted of the selected studies, using the complete ‘findings/results’ sections from the papers.

**Results:**

We included 48 qualitative studies representing the views of more than 3,400 older adults living at home in 11 Western countries. The QoL aspects identified in the synthesis were categorized into nine QoL domains: autonomy, role and activity, health perception, relationships, attitude and adaptation, emotional comfort, spirituality, home and neighbourhood, and financial security. The results showed that although different domains can be distinguished, these are also strongly connected.

**Conclusion:**

QoL can be expressed in a number of domains and related subthemes that are important for older adults living at home. The findings further support that the concept of QoL should be seen as a dynamic web of intertwined domains.

## Introduction

The need for care services for older adults living at home will increase in the coming years because of the ageing population and deinstitutionalisation [1–3]. More and more older adults will age in their own home and the majority of them has one or more chronic disorders [4, 5]. For many of these disorders, cure is not an option. However, care is required to manage these disorders and to provide assistance with daily tasks in order to enable older adults to age at home. Care and support are provided by informal carers as well as various formal care services like rehabilitation, nursing care at home, day care, mental health and general practice care [6, 7].

At the same time, care providers are confronted with care reforms, budget cuts and increasing regulations for national and local commissioning and audit procedures [8]. As a result, the accountability of care services becomes increasingly important and there is a need to establish the value of such services. One way to determine this value is by assessing the outcomes achieved at the client level [9]. Maintenance of QoL is one the most important outcomes of care services for older adults. Several international action plans on ageing endorse the importance of QoL [10–13] and international interest in the measurement of QoL of older adults is growing [14–17].

It is, however, not evident how QoL should be defined or how it should be assessed. The debate about the definition of QoL is conducted among researchers from various disciplines and overlaps with explorations of the concepts successful ageing, subjective well-being, life satisfaction and happiness [18]. A taxonomy of the conceptual development of QoL shows that a large set of QoL frameworks exist, and from this set it was concluded that “*QoL is inherently a dynamic*, *multi-level and complex concept*, *reflecting objective*, *subjective*, *macro-societal*, *and micro-individual*, *positive and negative influences which interact together*” ([19], p.46).

While numerous definitions and theories of QoL have been proposed, a systematic overview of the opinion of older adults themselves is missing. Knowing what older adults themselves find important in life, is necessary to align the goals of care services to their expectations. Also, knowing what quality of life is from the perspective of older adults themselves is necessary for assessing the content validity of existing QoL measures. Qualitative studies can help researchers and decision makers to understand what QoL means to older adults, and a considerable number of such studies has been done. However, these studies are largely neglected, which may be due to the fact that each of these studies has been done in a particular setting with a particular study population and particular point of view [20, 21].

In order to assemble a more comprehensive picture of QoL, the findings from multiple qualitative studies can be combined in a synthesis that provides a range and depth of meanings, experiences, and perspectives of participants across contexts [22]. The larger scope and rigor of a synthesis compared to an individual study also means that there is a greater potential to influence policy and inform practice [20, 23–26]. A recent systematic overview, including all relevant qualitative studies on QoL from the perspective of older adults, is lacking. Earlier reviews did not (exclusively) focus on qualitative research [19], or were not systematic and included only a limited number of studies [18].

A method that is considered appropriate to aggregate findings from a large number of qualitative studies is the thematic synthesis approach [22, 24, 26–28]. This approach uses thematic analysis techniques to bring together and integrate the findings of multiple qualitative studies by identifying the main, recurrent or most important themes [26]. The aim of the current review is to synthesize the findings of qualitative studies that explored what QoL means to older adults living at home.

## Methods

### Search strategy

A review protocol was developed according to the Preferred Reporting Items for Systematic Reviews and Meta-Analysis (PRISMA)-statement (www.prisma-statement.org). PubMed, Ebsco/PsycInfo and Ebsco/CINAHL were searched from inception up to 28 November 2018 (by KMvL and JCFK). The following terms were used (including synonyms and closely related words) as index terms or free-text words: ‘aged’ and ‘quality of life’ or ‘satisfaction’ and ‘narration’ or ‘understanding’ and ‘qualitative research’ or ‘focus groups’. The full search strategies for all databases can be found in the Supplementary Information (S1 File). Duplicate articles were excluded. All languages were accepted.

### Selection criteria

For inclusion in the review, articles needed to meet all of the following criteria: (1) focus on perceptions of QoL, (2) include older adults living at home as the main participants, (3) use of qualitative methodology, (4) conducted in a Western country, (5) published in English and (6) not focussed on specific patient groups. In order to avoid omitting research of potential value to the synthesis, qualitative methodology (criterion 3) was broadly operationalized as the use of open questions and a description of the findings in words rather than numbers [29]. Criterion 4 and 5 were used in order to keep the number of papers manageable. There was no restriction to publication year. Exclusion criteria were: participants from specific patient populations such as diabetes or cancer patients (as these studies tend to focus on disease-specific aspects of QoL), and participants living in residential facilities such as care homes, nursing homes or retirement homes.

### Selection procedure

The references from the different databases were imported into Mendeley [30], after which duplicates were removed. Each of two authors (KMvL and MSvL) screened half of the titles and abstracts to exclude articles that did not meet the inclusion criteria. In case of doubt, the article was included in the selection of papers potentially relevant to the review. Next, full texts were retrieved and further assessed for eligibility by both KMvL and MSvL, independently from each other. Disagreements were discussed by the two authors until consensus was reached. Reference lists of included studies were checked to identify additional relevant studies for the synthesis.

Since QoL is a dynamic and not strictly defined concept, we decided to use an inclusive policy regarding the focus on QoL. Researchers from different disciplines and backgrounds may have used various terms to describe aspects that contribute to quality to the life of older adults. Therefore, we included also studies using terms such as ‘life satisfaction’, ‘successful aging’ ‘living well’, ‘well-being’, and determined topical similarity by looking at the research purpose, the questions asked and the type of findings presented [21].

### Data extraction

All papers were read several times before and during analysis by two review authors (KMvL and MSvL). The following study characteristics were extracted by KMvL: information about the sample and sampling procedure, information about the data collection method and the methodological orientation or data analysis approach, information about the focus of the study and questions asked to participants, information about the theoretical frameworks that guided the development of questions or interpretation of findings, and the main conclusions from the authors.

The complete ‘results’ or ‘findings’ sections from the studies were seen as data for this review and entered verbatim into a Microsoft Excel database by KMvL or MSvL, simultaneously dividing the text into smaller but meaningful fragments. These fragments thus included quotes from participants as well as text written by the primary studies’ authors such as interpretations and clarifications of quotes, descriptions of participants’ responses and descriptions of the context in which responses were given. The labels, categories or themes under which the authors described the data were noted next to the specific fragments. Data from discussion sections (including more theoretical interpretations) was not extracted.

### Thematic analysis

We adopted the three stages of the analysis in thematic synthesis as described by Thomas & Harden [26]: free coding of the findings of primary studies; organisation of these free codes into related areas to construct descriptive themes; and development of analytical themes. The stages overlapped to some degree.

For the first stage of analysis, free coding, two authors (KMvL and MSvL) independently coded each extracted fragment according to its meaning and content. Within the fragments we identified QoL aspects (elements of older adults’ life that affected their QoL), and used these as codes to label the fragments. Each fragment was coded using at least one label, but more than one was possible as fragments could contain descriptions of multiple QoL aspects. The labels we used were primarily based on (description of) quotes of the participants rather than on labels used by the authors in the primary studies, since the categorization of similar quotes could be different, given that these authors had various disciplinary backgrounds or theoretical frameworks. After coding the first three papers, and subsequently after every two papers, KMvL and MSvL met to compare their codes, making sure that interpretations of codes were aligned. During the process of analysis, the coding scheme was discussed extensively and continuously, and adjusted and complemented if necessary. After 11 papers the definitions of codes were clear to both authors and only few new codes came up, so the remaining papers were divided between KMvL and MSvL to code them. Occasional new codes or updates of definitions were discussed, and applied to previously coded fragments.

In the second stage of analysis the free codes of all identified QoL aspects were combined or grouped in a total of 60 descriptive themes. In the third stage these were further categorised by KMvL and MSvL into 16 analytical themes after looking for similarities and differences between the descriptive themes and the fragments belonging to each descriptive theme. Each analytical theme was described by several subthemes. One analytical theme for example was ‘life philosophy’, which included fragments about ‘enjoying small things’, ‘staying positive’, ‘being interested’ etc. A draft summary of the findings across the studies was written for each of the 16 analytical themes by KMvL or MSvL, focusing primarily on the quotes of older adults in the fragments belonging to that theme. We subsequently reread the summaries and discussed the meaning of each theme and its relations with other themes. Disagreement or uncertainties were discussed and interpretations of the themes were validated with four of the other authors (JEB, RWJGO, FAvN, GAMW). During this stage, we identified similarities and strong connections between some of the analytical themes and decided to combine some of them. This final step resulted in 9 QoL domains described by 38 subthemes that cover all the QoL aspects we identified in the extracted fragements.

Finally, we examined to which extent the 9 QoL domains were covered in the data. We determined the coverage of a domain in each paper by looking at the total length of fragments that we categorized under that particular domain. Four ‘coverage’ categories were used: ‘not mentioned’, ‘briefly mentioned’ (in one to three sentences), ‘discussed’ (in a larger paragraph), and ‘discussed extensively’ (in more than one paragraph). We cross tabulated the papers and the domains showing the coverage in the cells, in order to get an overview of how consistently the QoL domains were mentioned across settings and subgroups of older adults.

## Results

**Fig 1:**
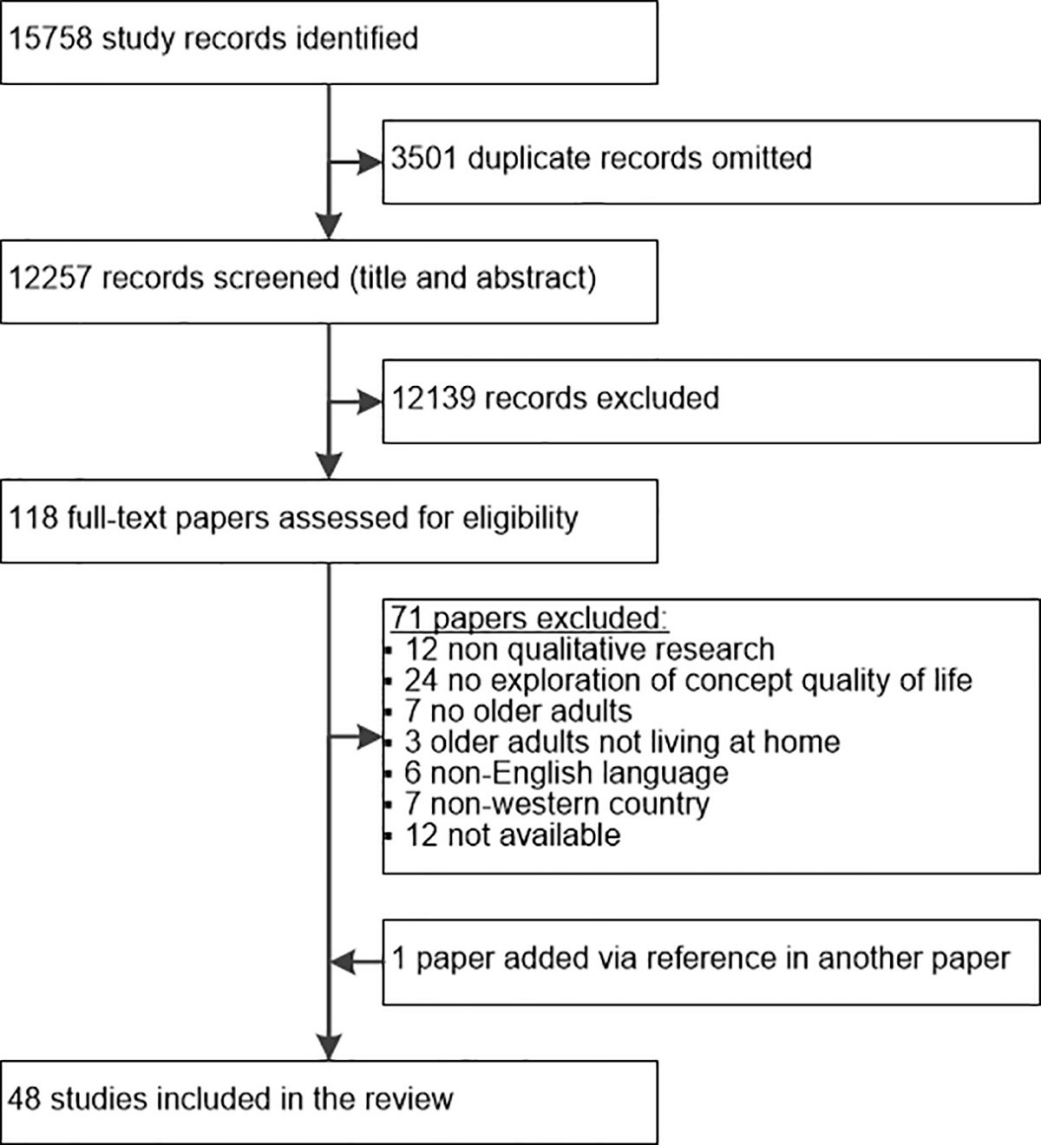
Flowchart for selecting studies.

The searches initially resulted in a total of 15,758 references, following elimination of duplicates this number was reduced to 12,257 references. After screening of abstracts, 12,139 papers were excluded because they did not meet the inclusion criteria. From the remaining 118 papers, full texts were retrieved and eligibility was determined by two authors. One paper was added via reference checking. Eventually it was agreed that 48 papers met our inclusion criteria. The details of the selection process are shown in Fig 1.

### Reviewed studies

Characteristics and conclusions of the 48 included studies are displayed in Table 1. The studies together included more than 3,400 older adults, who were living in 11 different countries. Most studies were conducted in the US (13 studies), Sweden (8 studies), the UK (7 studies) or Canada (7 studies). The samples were very diverse, including among others older widowed women living in rural and remote areas in Australia [31], foster grandparents from Missouri (US) [32], older adults receiving palliative care in Sweden [33] and older men residing in London (UK) [34]. Participants were most frequently recruited via care organisations and senior centres or via their participation in epidemiological cohort studies. In almost all studies either half of the participants or a majority of the participants was female. Reported mean ages ranged from 71 to 91 years. The most commonly reported reasons for excluding participants were minor or major cognitive impairment, not being able to communicate in a specific language, or less specific reasons for not being able to participate or give informed consent.

**Table 1:** Characteristics of studies included in synthesis.

Paper: Author year [ref] (country)	Sample and recruitment	Data collection method	Data analysis approach / methodological orientation	Focus of study	(Example of) questions asked to participants	Theoretical perspectives / frameworks that guided development of questions, codes or interpretation of findings	Conclusion
**Aberg** **2005** [35] **(Sweden)**	15 older adults (80+) with a diagnosed need of rehabilitation during a hospital admission, purposefully selected at the geriatric clinic	Qualitative interviews on three occasions: at the geriatric clinic, and 1 month and 6 months after discharge	Thematic framework approach	Factors perceived as important for life satisfaction	What is important for you to be able to do in order to be satisfied with life?	NR	Three themes emerged as important for life satisfaction: activity, independence and adaptation
**Andersson** **2008** [33] **(Sweden)**	17 older adults (75+) receiving municipal help and care and having a life-threatening disease or receiving palliative care, purposefully selected by nurses working in municipalities	Qualitative interviews with a narrative approach	Content analysis	Experience of aspects that bring about a good life in the last phase of life	Tell about your life situation especially what brought about a good life	NR	The experience was interpreted to be *Turning inwards to come to peace with the past*, *the present and approaching death while being trapped by health complaints*. Six categories embraced the experience of aspects that constitute a good life in the last phase of life: maintaining dignity, enjoying small things, feelings of ‘‘being at home”, being in the hands of others, trying to adjust, still being important for other people and completing life while facing death.
**Bergland** **2007** [36] **(Norway)**	282 older women (75+) randomly selected from census files local authorities, participating in a community-based randomized study	Writing down the answer on one open ended question	Grounded theory	What QoL means	Write down what the phrase QoL means to you		Significant dimensions of the quality of life highlighted in the current study relate to holism, a pragmatic approach of health, relationships, participation and activity, belonging and the social environment, and personal values. Feelings of continuity, power, and the ability to grasp meaning in their lives are cornerstones of the quality of life for elderly women.
**Bernardo** **2014** [37] **(Portugal)**	48 older adults (65+) selected from a health care centre registry	Semi-structured interviews	Content analysis	Perceptions of QoL	NR	Categories were created by taking consulted literature about QoL definitions into account	The older people who participated in the study described QoL as being healthy, having peace, living in harmony, feeling happy, being satisfied with life, and keeping oneself busy, whether with hobbies, volunteer service or work. It also meant preserving interpersonal relationships and receiving support from family, friends and neighbours.
**Black** **2015** [38] **(US)**	1) 51 community-dwelling older adults (65+), purposively sampled in a range of residential and recreational community venues 2) 216 community-dwelling older adults (65+), recruited via a range of media and in community venues with high volumes of older adult presence	1) Focus groups and 2) open-ended surveys	Content analysis	What matters most in the context of everyday community life	What matters most as you live your daily life in this community?	A reality-oriented perspective	Findings suggest five key themes, all underscoring the prominence of the continuation of self as one ages in the community: (1) Preserving and promoting health and well-being; (2) continuing living arrangement and lifestyle; (3) maintaining autonomy and independence; (4) engaging in meaningful social opportunities; and (5) accommodating community assets.
**Borglin** **2005** [39] **(Sweden)**	11 independent older adults (80+) living in their own home, purposively sampled via connections of the author	In-depth interviews	Interpretative hermeneutic phenomenological method	Experience and meaning of QoL	I am interested to hear about your thoughts, feelings, and experience of the good life/QoL and how this has influenced you.	NR	QoL in old age meant a preserved self and meaning in existence. The areas contributing to the experience of QoL were: life values, recollection of previous life, activities, health, significant others, material wealth, and home.
**Bowling** **2003** [40] **(United Kingdom)**	999 older adults (65+) living at home, randomly selected from British household addresses: The Quality of Life Survey	Face-to-face interview survey with open-ended questions	Content analysis	Definitions of and priorities for a good QoL	Thinking about your life as a whole, what is it that makes your life good/bad? What single thing would improve your QoL?	NR	Social relationships and health were judged to be the most important areas.
**Bowling** **2007** [41] **(United Kingdom)**	1) 999 older adults (65+) living at home, randomly selected from British household addresses: The Quality of Life Survey 2) subsample of 80 respondents for the validation of subthemes in in-depth interviews (purposively selected)	Mixed methods including open-ended survey and in-depth interviews	1) thematic categorisation 2) grounded hermeneutic methods + constant comparison	Main attributes that gave life quality (and underlying reasons)	Elicitation of respondent’s own descriptions of QoL (good and bad), their prioritisation of these things and how QoL can be improved	NR	The main reasons underlying the things people said gave their lives quality focused predominantly on: the freedom to do the things like they wanted to do without restriction; pleasure, enjoyment and satisfaction with life; mental harmony; social attachment; social roles; feeling secure.
**Bryant** **2001** [42] **(US)**	22 community-dwelling older HMO-members (60+) with a history of chronic conditions and high utilization, randomly selected from a group whose reported perceived health differed from that predicted by a regression model	Semi-structured interviews	Grounded theory-type methods	Factors that contribute to healthy aging	What they themselves believe describes and contributes to health/ well-being	NR	To these older people health meant going and doing something meaningful, which required four components: something worthwhile to do, balance between abilities and challenges, appropriate external resources, and personal attitudinal characteristics
**Bryant** **2004** [43] **(Canada)**	Older adults in 7 participating cities, more information NR	Focus groups and individual interviews	Each of the seven project sites carried out its own data analysis.	Perceptions of the influence upon their QoL	What are the events or situations that have diminished/ improved quality of life for you or for people living in your community? What things that affect your life could be influenced by governments?	Each of the seven project sites used their own framework, for example the concept of distributive justice, or an ecological approach	There was agreement across all locations as to the importance to seniors’ QoL of the following: access to information, health care, housing, income security, safety and security, social contacts and networks, and transportation.
**Carr** **2017** [44] **(Canada)**	42 community dwelling older adults (65+), purposefully sampled from a local Center for Seniors, a senior’s walking program, a senior’s exercise program, a local church and through snowball sampling	6 focus groups and 16 semi-structured interviews	Naturalistic inquiry	Factors that contribute to successful aging during different decades of older adulthood	What does it mean to age successfully? What do you think contributes to successful aging?	NR	Primary themes related to successful aging (staying healthy, maintaining an active engagement in life, keeping a positive outlook on life) were agreed upon by participants in all decades of older adulthood, while age-based differences existed among secondary themes.
**Cherry** **2013** [45] **(US)**	90 older adults (60+), drawn from the Louisiana Healthy Aging Study	Survey with 3 open ended questions	Content analysis consistent with grounded theory	Perceptions of longevity and successful aging	What is the key to living a long life? What do you look forward to the most? What advice or words of wisdom would you have for a young person today?	NR	Three major themes: (1) maintaining physical, mental and relational well-being; (2) living a healthy life; and (3) living a faithful life
**Dionigi** **2011** [46] **(Canada)**	21 older women (75+) with varying physical activity levels, purposeful selected from a seniors’ centre and retirement community	In-depth interviews	Inductive analysis; narrative content analysis	Meaning of ‘old’ and ‘successful aging’	Do you have any hobbies or regular activities that you enjoy? What does successful aging mean to you?	Stories were interpreted in the context of biomedical, psychosocial, and biographical approaches to successful aging	The findings particularly highlighted the multiple ways women make sense of their own aging and the extent to which their stories resist and reproduce cultural discourses of aging and gender. It appears that the more active the women, the more their definitions reflected key concepts in the biomedical model.
**Douma, 2015** [47] **(the Netherlands)**	66 older adults (65+), recruited through local gatekeepers	Interviews with ‘participant-generated word-clouds’	Content analysis	Subjective well-being in older age	Write down all aspects that you consider to be important for personal well-being	NR	15 domains were found based on participants’ conceptions of subjective wellbeing. The multidimensional domains of social life, activities, health, and space and place were most important.
**Duay** **2006** [48] **(US)**	18 healthy senior adults (59+) familiar to the researchers (convenience sampling)	Interviews with open ended questions	Constant comparative method	Perceptions about successful aging and the role of learning in the process of adapting to age-related changes	NR	NR	Successful aging involves engaging with others; coping with changes; and maintaining physical, mental and financial health
**Ebrahimi** **2013** [49] **(Sweden)**	22 frail older adults (65+) with diverse ratings of self-perceived health, purposefully selected from a sample who were included in a quantitative study after seeking emergency treatment in a hospital	Qualitative interviews	Content analysis	Influences on subjective experience of health	Can you describe a day/situation, where you experience health? What gives you a feeling of poor health? Please tell me more about your experiences, thoughts, and emotions / your everyday life.	Eriksson’s definition of health (endurable suffering) was used as guiding framework	To feel assured and capable was the main theme, which consisted of five subthemes: managing the unpredictable body, reinforcing a positive outlook, remaining in familiar surroundings, managing everyday life, and having a sense of belonging and connection to the whole.
**Everingham** **2010** [50] **(Australia)**	33 members (50+) of seniors’ groups, purposively selected and via snowball sampling	10 semi-structured interviews and 5 group interviews	Thematic coding	The meaning of aging well	What does aging well mean to you? What are the main issues that should be addressed to improve the lives of seniors in this community?	NR	Community perceptions of aging well are broadly consistent with the goals of national and international policy frameworks in focusing on 3 dimensions–health, social engagement, and security.
**von Faber** **2001** [51] **(the Netherlands)**	27 older adults (85+) participating in the longitudinal Leiden 85-plus Study, purposefully selected	In-depth interviews	NR	Perceptions about physical, social, and psychocognitive functioning and well-being	About the experience of growing old and being old, the perception of the concept of successful aging, and the role of health in successful aging	NR	Most elderly patients viewed success as a process of adaptation rather than a state of being. They recognized the various domains of successful ageing, but valued well-being and social functioning more than physical and psychocognitive functioning
**Fisher** **1992** [52] **(US)**	19 older adults (62+) recruited at a Senior Activity center	Open ended interviews	NR	Understandings of successful aging and life satisfaction	What successful aging and life satisfaction meant to them, what was necessary for each, and what prevented each.	NR	There were some overlaps in understandings of successful aging and life satisfaction, but there was one key difference. Respondents described life satisfaction in terms of past expectations and present circumstances, while successful aging was more oriented to strategies for coping in later life and maintaining a positive outlook
**Fisher** **1995** [32] **(US)**	40 older adults (61+), randomly selected from employees of a foster grandparent program	Open ended survey questions	Content analysis	Understandings of successful aging and life satisfaction	What successful aging and life satisfaction meant to them, what was necessary for each, and whether these concepts were relevant to their own aging experience.	Erikson’s concept of ‘generativity’ and Maslow’s self-actualization hierarchy were used to interpret parts of the findings	Successful aging and life satisfaction are two different, yet related dimensions of subjective well-being. Understandings of successful aging involved attitudinal or coping orientations nearly twice as often as those for life satisfaction. Life satisfaction appeared to represent basic needs, whereas successful aging corresponded more closely to higher order needs such as self-understanding, helping others, and feeling like one has made a difference.
**From** **2007** [53] **(Sweden)**	19 older people (70+) dependent on community care, purposefully selected by a professional care needs assessor	Two interviews, 2–3 weeks apart	Content analysis	Views about health and well-being	What health, ill-health, well-being and ill-being meant to them	NR	The opportunity to feel healthy was dependent both on the older peoples’ own ability to adjust or compensate, and on how the caregivers, relatives and friends in their environment could compensate for the obstacles the older people faced due to their disabilities.
**Fry** **2000** [54] **(Canada)**	37 older adults (58+), subsample of households participating in a community-based study	In-depth interviews following a open-ended survey	Content analysis	Considerations, priorities and concerns regarding QoL	What individual domains are the most important to your QoL? What specific concerns do you have about the QoL of your life?	NR	The majority of respondents has clear demands for autonomy, control and independence in making decisions, including the decision to terminate life.
**Gabriel** **2004** [55] **(United Kingdom)**	80 older adults (65+) living at home, purposively selected from respondents to a quantitative survey (The Quality of Life Survey)	In-depth interviews using semi-biographical interview techniques, repeated with half of the sample 1 year later if changes were reported	Thematic coding	Perspectives on QoL	What they thought of when they heard the words ‘QoL’, to describe their QoL, what gave their lives quality and what took it away, how it could be improved and what would make it worse.	NR	The main QoL themes that emerged were: having good social relationships, help and support; living in a home and neighbourhood that is perceived to give pleasure, feels safe, is neighbourly and has access to local facilities and services including transport; engaging in hobbies and leisure activities (solo) as well as maintaining social activities and retaining a role in society; having a positive psychological outlook and acceptance of circumstances which cannot be changed; having good health and mobility; and having enough money to meet basic needs, to participate in society, to enjoy life and to retain one’s independence and control over life.
**Gilbert** **2012** [56] **(US)**	10 older adults (80+) living in an urban area, purposefully sampled, known by researchers	Semi structured interviews	Phenomenological approach	Perceptions of facilitators and barriers to healthy ageing	What do you do to stay healthy? What are the factors that help you remain active? What are the barriers to remaining active?	Dorthea Orem's Self Care Nursing Theory	Three themes were identified as facilitators to healthy ageing: taking care of self, meaningful activity; and positive attitude. Barriers to healthy ageing identified were: giving up or giving in; environmental limitations; and the ageing process.
**Grewal** **2006** [57] **(United Kingdom)**	40 older adults (65+) living in private households, purposefully selected from a respondents to a general population survey	In-depth interviews	Thematic approach	Perceptions about QoL	What was important to them, what they enjoyed, got pleasure from, or valued in their lives. And, what is it about (factor) that is important to you, how does it make a positive contribution to your live?	Results were interpreted using work from Hyde, Higgs and colleagues that distinguishes attributes of QoL from the influences upon it; and Sen’s functioning and capability approach	Initial discussions tended to concentrate upon factors influencing QoL including activities, relationships, health, wealth and surroundings. Further probing and analysis suggested five conceptual attributes: attachment, role, enjoyment, security and control. The data also suggested that QoL was limited by the loss of *ability* to pursue these attributes.
**Hendry** **2004** [58] **(United Kingdom)**	10 older adults (70+) recruited via day centres	Semi-structured interviews	Interpretative phenomenological analysis	Understandings of QoL	Questions focusing on five themes (physical health, psychological well-being, social relationships, environment and choice and control) and any aspects of QoL not covered yet	NR	Older people’s understandings of quality of life are not readily measurable and should be viewed in terms of phenomenological experience: 1) when offered to give a general picture of QoL, people do not segment their lives into component parts; 2) participants often compared their own experiences to those of others (contextual experience); 3) participants did not share the experience of a linear effect of aspects of aspects on QoL 4) perceived QoL varies on an ongoing daily basis and participants choose which aspect of QoL to make public.
**Hinck** **2004** [59] **(US)**	19 older adults (85+) living alone in their own home in a rural area, selected via purposive and network sampling techniques	In-depth interviews, at least 3 per participant	Interpretative phenomenology	Life experiences	Talk about what is meaningful to you. Tell me what yesterday was like	NR	Remaining at home is a strong value of even the oldest-old people. Although they might be managing day to day, their ability to continue safely at home might be tenuous and could easily be upset by illness or injury. Participants were creative in changing their environment and everyday practices and patterns to be able to complete most desired activities.
**Hörder** **2013** [60] **(Sweden)**	24 community-dwelling older adults (77+) recruited from a health promotion intervention	Open interviews	Content analysis	Perspectives on successful ageing	Tell me what successful ageing means to you	NR	Successful ageing can be seen as a preserved self-respect through ability to keep fear of frailty at a distance. This embraced the content of four categories: “having sufficient bodily resources for security and opportunities”, “structures that promote security and opportunities”, “feeling valuable in relation to the outside world”, and “choosing gratitude instead of worries.”
**Kalfoss** **2010** [61] **(Norway)**	20 older adults (60+) living in the community, selected via senior organisations or nurses when acutely hospitalized or attending ambulatory care	Focus group interviews	Thematic content analysis	Issues of importance to QoL	Think about what the phrase QoL brings to mind. What issues contribute positively or negatively to your QoL?	After analysis, themes were compared with Lawton’s conceptualisation of QoL	Many valued aspects of human existence were found to affect QoL, and results lend empirical support to many of the themes appearing under Lawton’s four sectors.
**King** **2012** [62] **(US)**	62 community-dwelling older adults with late-life disability, recruited from a senior care program (On Lok Lifeways)	Semi-structured interviews	Grounded theory (constant comparative analysis)	Factors that contribute to QoL	About participants’ daily lives, including positive and negative aspects and descriptions of daily activities	NR	Participants described a range of factors in four domains (physical, psychological, social, spiritual) that contributed to their QoL. Dignity and a sense of control were central factors that had the strongest effect on QoL by allowing participants to build autonomy and self-worth.
**Laditka** **2009** [63] **(US)**	396 older adults from ethnically diverse groups living in the community, recruited within The Healthy Brain Project via a research network	Focus groups interviews	Constant comparative method	Views about ageing well, in the context of cognitive health	Please tell us about someone who you think is ageing well.	NR	There were notable race/ethnicity differences in perceptions of aging well. To promote cognitive health among diverse populations, communication strategies should focus on shared perceptions of aging well, such as living to an advanced age with intact cognitive function, having a positive attitude, and being mobile.
**Levasseur** **2009** [64] **(Canada)**	18 community-dwelling adults (60+), theoretically sampled based on disability level and QoL evaluation in a quantatitive study	Two face-to-face semi-structured interviews (one week apart)	Phenomenological method	Perceptions and lived experiences about QoL in regards to personal factors, social participation and environment	In your own words, tell me what QoL means to you? What had the most positive/ negative effect on QoL? How do everyday activities influence your QoL?	Themes were inspired by a disability and functionings model and Dijkers’ conceptualisation of QoL	These results point up the importance of considering perceptions about personal factors, social participation and environmental factors in older adults’ QOL.
**Llobet** **2011** [65] **(Spain)**	26 older adults (75+) selected from a home health care service database, representative in age and gender	Face-to-face interviews with 4 open-ended questions	Content analysis, grouped into categories	Elements composing QoL	How do you define QoL? What are reasons for your QoL rating? What are aspects related to satisfaction with life?	Results are explained with Role Theory and Engagement Theory	Main reasons for a good perception of QoL were health, family and social relationships, and the ability to adapt.
**Lorenc** **2012** [66] **(United Kingdom)**	37 older volunteers (61+) from community voluntary organisations, during a ‘participant engagement event’	Focus groups	Content analysis	Perceptions and experiences of well-being (and decision making regarding complementary and alternative medicine)	Perceived meaning of well-being, changes in well-being since the group last met, and factors influencing well-being	Content analysis was partly informed by existing literature	“Keeping going” is important for older people. Five themes emerged: physical well-being, impact on activity, emotional issues, community and health services, and keeping positive.
**Lysack** **2002** [67] **(US)**	23 Caucasian and Afro-American community-dwelling women (85+) as exemplars of ageing well, identified via like-aged community peers	In-depth ethnographic interviews	Constant comparative method	Personal meanings of ageing and well-being	Questions to identify what growing older was like and what it meant to participants.	Interviews were analysed through the combined theoretical perspective of symbolic interactionism and continuity theory	Personal competence in the ‘‘feminine sphere” is key to understanding older women’s health beliefs and behaviours in late life. Findings also point to the importance of occupational competence as a predictor of well-being in late life.
**Milte** **2014** [68] **(Australia)**	21 older adults (64+) attending outpatient day rehabilitation services (incl therapy gym and hydrotherapy sessions)	Semi-structured focus groups (including ranking exercise)	Mixed methods; for qualitative part structured content analysis was used with thematic coding procedures	Perceptions of QoL	Tell me about what QoL means to you? / Why did you rank this item as most/least important?	Existing QoL instruments used (ASCOT, OPQoL) for ranking exercise, on which the group discussion was based	Older adults value both health and social domains as important to their overall QoL.
**Moore** **2006** [69] **(Canada)**	11 older adults (65+) living in their own homes, lodges and senior complexes, recruited via a written invitation by colleagues of the authors	Narrative inquiry via in-depth interviews	Phenomenological reflection	Experience of meaning and purpose in life	As you reflect back over your life, what are meaningful, important experiences for you?	NR	It is in continuing to have a rich and satisfying life, even if it meant struggling a bit that seemed to contribute to a sense of meaning and purpose in life for the participants in this study.
**Murphy** **2009** [70] **(Ireland)**	122 older people with 6 types and different onset of disability, living in the community, purposeful selected	Interviews	Informed by grounded theory	Determinants of QoL	NR	Data collection was complemented by findings from international literature	We identified QoL factors that were important to older people with a disability, and these were consistent across groups, regardless of type of disability. ‘Living well’ was conceptualized as the core category.
**Nilsson** **1996** [71] **(Sweden)**	87 older adults (75+) without severe somatic or psychiatric disorders, living in their own homes, participating in a multidisciplinary longitudinal study (Kungsholmen Project)	Structured interviews with standardized and open questions	Content analysis	Characteristics of QoL	What does QoL mean to you?	The Finnish sociologist Erik Allardt’s definition of QoL was chosen as the conceptual framework for the study	The concept of QoL has many dimensions. A definition like Allardt’s is too static and does not cover all aspects of the elderly’s QoL. To sum up the characteristics of the QoL in old age, it can be stated that the emphasis is not on material things and the elderly’s own persons but on contentment and a peaceful life, independence and health as a resource for this personal integrity in terms of moral qualities, and a caring attitude.
**Nosraty** **2015** [72] **(Finland)**	45 community-dwelling older adults (90+), invitation sent to every fifth woman and man born in 1921–22, living in the city	Life-story interviews	Thematic analysis with an inductive approach	The meaning and content of good and successful aging	What do you think constitutes a good old age? What do you need in order to experience a good old age? What things are associated with it? And what do you think a good old age is?”	NR	Good health is important, but more in the sense of being pain-free than of being disease-free. Social and cognitive aspects seem to be more important than physical health. The important things for our nonagenarian respondents were to continue living independently, preferably in their own homes, and to have a quick and easy death rather than being institutionalized.
**Prieto-Flores** **2010** [73] **(Spain)**	24 older adults (64+), purposively selected at public day care centers and public seniors’ centers	Semi-structured interviews	Grounded theory	Connections between the subjective experience of health and other significant Qol domains	Around perceptions of aging, health, QoL, and health and social care	NR	Four major categories were identified: (a) adaptation to the limits of health in aging; (b) subjective health and QoL in aging: seeking a balance; (c) the experience of place in centers for older people; and (d) a central category, health and family interrelated dimensions of QoL in old age.
**Puts** **2007** [74] **(the Netherlands)**	25 older frail and non-frail community-dwelling adults (65+), theoretically sampled based on 8 frailty markers from the Longitudinal Aging Study Amsterdam	Semi-structured interviews	Grounded theory	Meaning of QoL	E.g. What is the first thing that you think about when you hear the term QoL? What is important for your own QoL, and why?	Topic guide was based on a literature study on QoL	Five themes emerged: (physical) health, psychological well-being, social contacts, activities, and home and neighborhood
**Reichstadt** **2010** [75] **(US)**	22 community-dwelling adults (60+), purposively selected at retirement communities, a low-income senior housing complex, and a continued learning center	Qualitative interviews	The method of ‘Coding Consensus, Co-occurrence, and Comparison’ (Grounded theory)	Perspectives on successful ageing	E.g. How would you define successful aging? What is important to aging successfully? What are your suggestions on how to age well?	NR	Two primary themes were identified as key to successful aging–i.e., self-acceptance/self-contentment (with sub-themes of realistic self-appraisal, a review of one’s life, and focusing on the present) and engagement with life/self-growth (with sub-themes of novel pursuits, giving to others, social interactions, and positive attitude). A balance between these two constructs appeared critical.
**Richard** **2005** [76] **(Canada)**	72 older adults (55+) living in an urban environment, recruited from purposefully selected seniors’ groups and community organisations serving older adults	8 focus groups	Descriptive analysis	Factors affecting QoL	On factors related to or affecting QoL, measures to improve QoL and the role governments can play in the QoL of older adults	The ecological model of McLeroy et al. was chosen as an organising framework for categorisation of factors	A broad range of issues were discussed. The most salient themes were health and independence, financial security, social integration, health care services, housing, accessibility of community services, and decision-making power.
**Romo** **2013** [77] **(US)**	56 community-dwelling older adults (55+) with late-life disability from different race/ethnic groups, recruited from a senior care program (On Lok Lifeways)	Semi-structured interviews	Grounded theory (constant comparative analysis)	Meaning of successful ageing	What comes to mind when hearing the term ‘succesful ageing’? What does it mean to be old? Do you feel you’ve aged successfully? Do you feel old?	NR	An overarching theme was that aging results in *Living in a New Reality*, with two subthemes: *Acknowledging the New Reality* and *Rejecting the New Reality*. Participants achieved successful ageing by using adaptation and coping strategies to align their perception of successful ageing with their experiences. Themes were common across race/ethnic groups but certain strategies were more prominent among different groups.
**De la Rue** **2003** [31] **(Australia)**	5 rural older (65+) widowed women, voluntarily recruited based on a homogenous sampling strategy	Repeated in-depth interviewing based on a life history research approach	Thematic analysis	(Influence of the geographical location on) the meaning of health and well-being	Tell me about your life here on this property	Social constructionism and socio-environmental theory of gerontology provided the philosophical boundaries to the central research question	The informants’ health and well-being were profoundly influenced by the geographical location of living on the land
**Thomas** **1989** [34] **(UK)**	20 older men (70+) residing in England, purposively selected (to match an Indian sample) from organisations serving the elderly (50 participants interviewed; analysis based on 20 participants);	In-depth open ended interviews	Hermeneutical approach	Life satisfaction / subjective well-being	About themselves, their past and their present situation (things they enjoy and dread, attitudes toward pain, pleasurable experiences, etc.)	NR	The samples (Indian and English) differ in overarching themes and their level of life satisfaction. The dominant theme for the English sample is dread of incapacitation, of becoming useless and dependent. The term that best describes these men is *stoic acceptance*, if not resignation.
**Tollen** **2008** [78] **(Sweden)**	22 older adults (65+) with disabilities, purposefully selected from persons who applied for (but not yet started) day care rehabilitation	Qualitative interviews	Phenomenography	Everyday life experiences	‘I would like you to tell me about your everyday life, what you do and how you experience your situation’.	NR	Disengagement in activities and social contacts resulted in feelings of resignation and dejection for some participants, while others delegated tasks as a satisfactory alternative. Participants also described how activities and social contacts continued, albeit in different ways, and being active and socializing gave feelings of pleasure and a sense of belonging. While receiving help was experienced as valuable, it also increased the fear of becoming dependent.

NR = not reported

Nine studies used focus groups or group interviews for data collection, five studies were based on open questions in a survey, and the remaining studies used individual interviews. The method of analysis consisted of content analysis or thematic coding, grounded theory analysis, and phenomenological or hermeneutical approaches.

### Quality of life domains

We categorized the QoL aspects included in the extracted data into nine QoL domains: ‘Health perception’, ‘Autonomy’, ‘Role and activity’, ‘Relationships’, ‘Attitude and adaptation’, ‘Emotional comfort’, ‘Spirituality’, ‘Home and neighbourhood’, and ‘Financial security’. An overview of the domains and subthemes is shown in Table 2. Each domain is further described and illustrated with quotes in the section below.

**Table 2:** Domains and subthemes of quality of life.

Domains and subthemes	Description
**Health perception**	**Feeling healthy and not limited by your physical condition**
- [Physical conditions and symptoms]	- Not suffering from physical, mental and cognitive symptoms or disorders
- [Point of reference]	- Feeling healthy compared to prior health status or that of others
- [Health as an ability]	- Not being limited by your health
**Autonomy**	**Being able to manage on your own, retaining dignity and not feeling like a burden**
- [Independence]	- Being able to manage on your own and do what you want
- [Control]	- Being able to choose what you want
- [Burden]	- Not feeling like a burden to others
- [Dignity]	- Being able to retain dignity by focusing on things that one can do
**Role and activity**	**Spending time doing activities that bring a sense of value, joy and involvement**
- [Control over time]	- Having the freedom to organize your time
- [Keeping busy]	- Having something to stay occupied and keep you from feeling bored
- [Valuable activities]	- Doing activities that bring joy or meaning to life
- [Staying connected]	- Staying mentally active, up-to-date and in touch with the world around you
- [Helping others]	- Feeling able to contribute to society and making a difference
- [Achievements]	- Being proud on (and achieving a sense of identify from) current and former achievements
- [Self-worth]	- Feeling valuable and comfortable in your own skin
**Relationships**	**Having close relationships which makes you feel supported and enable you to mean something for others**
- [Close relationships]	- Having (and keeping) valued relationships
- [Family]	- Enjoying bond with partner and/or (grand)children
- [Experiencing support]	- Experiencing that people care for you and care about you
- [Love and affection]	- Experiencing a sense of belonging and intimacy, being loved and appreciated
- [Reciprocality]	- Having the possibility to help and support others
**Attitude and adaptation**	**Looking on the bright side of life**
- [Positive attitude]	- Being positive and making the best out of life
- [Acceptance]	- Being able to accept what you cannot influence
- [Changing standards/expectations]	- Being able to put your situation into perspective (cognitively minimizing effects of deteriorations by lowering standards and comparing yourself favourably to others)
- [Changing behaviour]	- Being able to change habits, do things differently or with assistance from others/aids
**Emotional comfort**	**Feeling at peace**
- [Calm vs worried/anxious]	- Having peace of mind (not feeling worried or anxious)
- [Happy vs sad/depressed]	- Being happy (not sad or depressed)
- [Loneliness]	- Not feeling lonely or isolated
- [Reminiscence]	- Not feeling troubled by past experiences
**Spirituality**	**Feeling attached to and experiencing faith and self-development from beliefs, rituals and inner reflection**
- [Being religious]	- Having religious beliefs, faith in God
- [Being spiritual]	- Being on a quest for meaning, self-development and awareness
- [Religious activities]	- Being involved in religious activities or a religious community
**Home and neighbourhood**	**Feeling secure at home and living in a pleasant and accessible neighbourhood**
- [Meaning of home]	- Having a home that provides privacy and comfort
- [Living at home]	- Living as long as possible in your own home
- [Safety]	- Feeling safe and secure at home and in the neighbourhood
- [Neighbourhood]	- Living in a pleasant neighbourhood with friendly neighbours
- [Accessibility]	- Being able to access and transport to important areas in the neighbourhood
**Financial security**	**Not feeling restricted by your financial situation**
- [Sufficient money]	- Having sufficient money to meet basic needs
- [Financial freedom]	- Having the financial freedom to enjoy life
- [Materials and conditions]	- Having material resources to feel comfortable and independent

#### Health perception: Feeling healthy and not limited by your physical condition

Health is mentioned as a necessary and sometimes even paramount element of QoL, eg: “*Above all*, *being healthy is the most important thing to have quality of life*, *the rest comes as an extra*” ([37], p.76). The perception of health was partly determined by the extent to which older adults felt fit and active or suffered from **physical, mental and cognitive disorders**. Troublesome symptoms, functional limitations and side effects from medication (such as poor balance, poor memory, pain, vision loss and fatigue) significantly decreased their QoL. “*Blindness is the one thing and then I was diagnosed with diabetes and then I had a heart operation*. *I’m still walking around and I still enjoy playing music so in that way I’m blessed but in other ways I’m not* …” ([77], p.944).

The perception of health is also determined by the **point of reference** used; older adults compare their health for instance often with that of others their age. The experience of health was therefore described as a relative phenomenon [36]: it is experienced and evaluated according to what one finds reasonable to expect, given one’s age, history, medical condition, and social situation. For example, some older adults find declining ability frustrating "*I cannot do what I did ten years ago*, *and I get very angry* … *I get disgusted with myself*" ([42], p.934), while others were more accepting, saying that other people are worse off, or that pain, fatigue and illness were to be expected in old age. This explains why older adults may still perceive their health as ‘good’ despite chronic diseases, illnesses and frailty [59, 60, 67]. Some older adults are committed to improve their QoL by trying to influence their health with a positive attitude and an active lifestyle.

Health was described as important because it is the basis for many other QoL aspects: *“What mostly controls it is your personal health*. *That determines what you can do*.” ([72], p.55). Good health appeared to facilitate the **ability** to carry out meaningful activities, to take care of yourself, to perform household tasks, to get out, communicate and participate. Murphy et al. [70] noticed that especially as participant’s physical functioning declined older adults started to redefine health in terms of abilities rather than absence of illness, e.g. “*Health is to a great extent being able to look after yourself*” ([49], p.292). Only when older adults were severely restricted in their ‘going and doing’ [42], they experienced poor health. Such experiences were connected with negative emotions of sadness, anxiety and sorrow [53].

#### Autonomy: Being able to manage on your own, retaining dignity and not feeling like a burden

Many older adults mention the desire to stay **independent** as long as possible:

“*If I recover from this and can take care of myself*, *then I shall be satisfied*. *It's important not to have to trouble others*, *and to be able to carry out the duties I can”* ([35], p.1116).

Participants say that being independent enables them to experience a sense of freedom and to enjoy life, by being able to socialise, to go outdoors and to do what you want. As Bryant et al. [42] noticed, respondents spoke with pride or pleasure of their independence, saying for example ‘we can take care of ourselves’ and by giving examples of everything they still do. Several participants indicated that the worst imaginable situations would be to become completely dependent on others or to end up in a nursing home.

Carrying out activities and daily routines independently contributes to a sense of **control**. For example, doing household task without help implies that older adults can decide themselves when and how it is done; i.e. to one’s own specifications. Older adults emphasize that being dependent, or old, does not mean that one is unable to make decisions or express wishes:

“*When it comes time for decisions concerning my health*, *my money*, *and how and where I want to live and die*, *I am fully in charge of myself and my family… Just because a man is getting old*, *and a bit slow does not mean that others can force their way into his life and tell him what’s good for him*.” ([54], p.375).

When you are used to manage on your own without having to take into account another person’s time and availability to help, it can be difficult to accept and acknowledge the need for help. Older adults do not want to be a **burden** to others. Although they value they value the assistance and support they receive, they often indicate that they are afraid to be(come) a burden to others. Some therefore withdraw from contact with friends and family “*You don’t want to spoil theirs [enjoyment] and be a nuisance to them*” ([53], p.283) or even rather prefer the thought of passing away [35, 42].

Authors of the included studies remarked that feelings of being a burden, guilt and embarrassment due to dependency affect older adults’ self-image. This may be the reason why, rather than acknowledging to be dependent, many older adults characterised themselves as independent by emphasizing the things they can still do [77, 78]. Focusing on remaining abilities, personal care and appearance helps to retain **dignity**, and when care is needed: being helped and treated with respect for one’s personal identity, wishes and values. From et al. [53] and Milte et al. [68] noticed that especially partners and relatives can help the older person to preserve an experience of dignity as they are well aware of their habits and are sensitive to their wishes: *“[My husband] always said ‘I’m so lucky’ because I was there to care for him and that gave him dignity and I feel that that dignity in our life is very important*” ([68], p.80).

#### Role and activity: Spending time doing activities that bring a sense of value, joy and involvement

Older adults living at home value the **control over their time**, i.e. the freedom to spend their time as they prefer: *"The most positive thing*? *I feel free* … *there's no rush* … *I want to take advantage of what I have to do before I can't do it anymore*." ([64], p.e96). For one respondent, this freedom was even the definition of well-being: well-being means “*to get up and do as much as you want to when you want to”* ([42], p.933).

In almost all studies, respondents report that it is important for their wellbeing to **‘keep busy’**, ‘keep active’ and ‘have something to do’ in order to avoid boredom and sink into apathy. For some however, particularly those with severe limitations, time passes by slowly: “*Yes*, *obviously*, *you are lying and thinking and sometimes dozing*, *even sleeping sometimes*. *There's nothing else to do*. *[…] I do not know what to call it*, *do not have a name for it*, *but*: *deadly boring*.” ([35], p.1120).

Respondents mentioned a broad range of **valuable activities** that brought joy or meaning to their life, including hobbies, mental and physical activities or more socially oriented activities, such as going out, travelling and participation in group activities. Indicative is the following quote: “*I love the theatre*, *I love the cinema* … *If it's just to go off for a day somewhere and have a meal in a pub* … *And I think that's very essential*, *it's just the simple pleasures of life really*" ([57], p.1897).

Older adults indicate that activities such as reading the newspaper, learning new things and keeping in touch with family and friends also help to **stay connected** with the world around them. They value feelings of belonging, participation and having a role in society, although they sometimes feel excluded: “*It’s unfortunate that a lot of your family or other people feel that*, *once you’re old*, *you don’t know anything anymore and you’re just kind of in the way*.*”* ([62], p.574)

In order to feel valued and to gain a sense of purpose, older adults mentioned that they find it important to continue **helping others**, for example by volunteering, babysitting or caring for sick relatives. By doing so they feel they contribute to society and fulfil a role: *“I think the quality of life is being involved and having a part to play*. *I think if you lose your role in life then you start getting depressed*, *I think it is very important to be needed for whatever reason*, *and … kind of have self-worth or something and know that people think you are worthy*.*"* ([55], p.685]).

Current and former roles and **achievements** were often mentioned by older adults as something to be proud of. Specific examples for women were given in the study of Lysack & Seipke ([67], p.134): “*I feel as though I accomplish things when I can keep my own yard and keep my own house"*. For many respondents it feels important to continue the activities they have always done, as these are an important part of their identity.

Thus, older adults’ **self-worth** is closely related to the activities they are able to do and achievements and roles in current and earlier life. The effect of limitations on one’s self-image is for example described as follows: “*Yes*, *I think it’s meaningless when I obviously can’t be active in any way… You don’t feel like a real human being in some ways*, *more like a vegetable or something*” ([35], p.1115). Respondents mention that it is important to have a positive picture of yourself, but that their self-worth is sometimes threatened by the feeling of being perceived as a nuisance in society.

#### Relationships: Having close relationships which makes you feel supported and enable you to mean something for others

Social contacts are seen as essential for QoL by older adults, they help to avoid loneliness. Especially **close relationships** are valued, as the quality of contact is most important: “*Togetherness is understanding and trusting someone else*. *Being able to talk to someone about things you wouldn’t speak to others about and them doing the same*.*”* ([53], p.282).

Many quotes show the importance of relations with **family** members; to provide help, support and love, and give joy and meaning in old age; e.g.: “*How can I put this*, *the older you get*, *the more you focus on your own family and your children*. *[…]*. *Yes*, *you’re there for each other*.*”* ([74], p.269). Hörder et al [60] remarked that feeling loved by close relatives and caring about them can keep older adults from worrying and distract their attention. Especially partners and (grand)children are a source of joy and support, although the bond may also bring worries, conflict and sorrow. Fear of violence and abuse were only marginally reported [37, 54, 76].

**Experiencing support** (both practically and mentally) in personal relationships was a recurring theme, and older adults talk about how they appreciate being looked after by people that care about you, having someone you can call if you have any problems, and being encouraged in times of setbacks. Some older adults who lacked close relationships expressed the wish for support: “*I'd just want friendship*, *some compassion*, *understanding*, *empathy*.” ([42], p.936). A bond with pets can provide support as well [40, 64].

Besides providing support, close relationships can be a source of **love, affection** and appreciation: “*Being in the company of my dear ones gives me a sense of belonging*. *It’s nice to know that someone cares*, *and that they in turn can count on me when they need help or someone to talk to […] Above all*, *I want to love and be loved*.” ([36], p.43). Sexuality and intimacy were only briefly mentioned in the studies by Kalfoss [61] and Bryant et al. [42].

As shown in above quote, older adults want **reciprocal relationships** and mean something for others as well. Keeping relationships equitable may be especially important to maintain self-worth and diminish feelings of being a burden.

#### Attitude and adaptation: Looking on the bright side of life

Older adults strongly believe that a **positive attitude** helps to have a good QoL. Older adults who adopted such a life philosophy described their motto as: stay positive, enjoy life, be happy with small things, make the best of life, and maintain your humour, optimism and curiosity. They emphasize one should not feel sorry for oneself, complain all the time or just sit down and do nothing.

In many ways, ageing relates to ‘living in a new reality’ [77]. In order to maintain a feeling of wellbeing, older adults state that it is essential to accept, adapt to and cope with life changes inherent to ageing, such as retirement, slowing of pace, declining health status (of partner) and loss of loved ones. Inability to adapt may result in feelings of sadness and despair. Some older adults even equate QoL with the ability to adapt:

“*20 or 30 years ago*, *I might have listed some of the usual terms associated with the expression “good quality of life”*: *good health*, *an adequate income*, *a reliable social network*, *good friends*, *and an enjoyable and encouraging job*. *Today*, *at the age of 86*, *my definition is different*. *With a growing absence of those pleasures*, *I now see a good “quality of life” as the ability to adapt to an increasingly difficult situation without letting problems interfere with gratitude and joyfulness; to experience fine literature and music*, *and as far as one can manage*, *to be useful and encouraging towards other people*. *In other words*, *a good “quality of life” is the ability to endure no matter what*." ([36], p.43).

Older adults used several ways to adapt to difficult situations, including deliberate and unconscious processes. **Acceptance** is the strategy most often mentioned. Respondents recommend to make the best out of each situation and to take life as it comes by accepting what happens to them: “*You never do get over it but if you can accept it you can start taking the steps*, *you’ve got to …life’s got to go on*, *hasn’t it*?*”* ([55], p.683).

Some more unconscious coping processes that were identified include minimizing and denying limitations, laughing about difficulties, emphasizing abilities and skills that one had in the past, and **adjusting one’s standard** of what is acceptable or important. A woman, who used to care a lot about looking representable and wearing panty hoses, now says, “*But it was so much trouble*. *[*..*] So I thought*, *what the heck*, *I’ll just go bare legged*. *I don’t care what people think about it*” ([59], p.787). Older adults put their situation into perspective by comparing their situation to others, or to what is normal or expected at their age. This could entail either ‘upward comparisons’ (e.g. inspired by the strength, will and optimism of others) or ‘downward comparisons’ (e.g. feeling grateful when considering your health better than that of your peers).

Older adults also adapt and maintain their QoL by **changing their behaviours** and habits. Some respondents, for example explain that they decreased their level and duration of activities in order to prevent fatigue or deterioration of their health: “*You have to take a rest and then you can do it again*.*”* ([39], p.205). They also said to avoid risks and to be more cautious or attentive when carrying out tasks, and to seek more assistance from other persons, technology, aids and equipment. Some even anticipated to future limitations, such as moving to live near children or investing in a good physical or cognitive condition.

#### Emotional comfort: Feeling at peace

Older adults indicated that they wish to feel **calm**, content, free from worries, in harmony with life and to have peace of mind. However, for some, these feelings are hampered by stress and worries about loved ones, low income, health and independence: “*The worries that drag around with you is like a weight you put in the trunk of your car that slows you down*.” ([64], p.e95). Some older adults express severe levels of stress, and report to feel frightened, fear the future or have anxiety. This severely impacts their QoL.

Quite some respondents mentioned (often in relation with their positive attitude) to be **happy**, enthusiastic, embrace life, and appreciate or enjoy small things. Others report to feel sad, downcast, hopeless or even depressed. A participant in the study by Bernardo et al. ([37], p.77) said: “*Nothing has a positive impact on my quality of life… loneliness*, *sadness*, *that’s what has damaged my quality of life the most*.”

**Loneliness**, usually as a result of bereavement or moving into a new community, has a strong negative impact on older adults’ QoL. Borglin et al. ([39], p.211) pointed out that the loss of a partner implies immense sorrow and a feeling of having lost an important part of oneself: “*The loss is indescribable*, *when you have been together for so many years*, *but that is why I say that I have lived my life*, *for the fact is that I’m not living any more*, *I only exist*.” Losing connections with friends and family members is difficult as well: “*My best friends are all gone… and I feel that is one of the worst parts of getting old and surviving*: *you have to bear the pain of seeing them go*.” ([42], p.935).

Lastly, the **reminiscence** of negative and positive past experiences can influence current feelings. Some memories are painful, e.g.: “*These memories*, *they never go away*. *No matter what pills you take*, *it doesn’t take away the burden that I’ve been carrying right up till today*” ([72], p.53), whereas others bring joy, or feelings of accomplishment and gratefulness.

#### Spirituality: Feeling attached to and experiencing faith and self-development from beliefs, rituals and inner reflection

**Being religious** or spiritual can support older adults in accepting disability or psychological distress, in coping with changes and being satisfied with life: “*It helps me to cope with happenings*, *incidents and so on*, *which would seem to be absolute tragedies to other people”* ([57], p.1895), and *“I just am so thankful for what the good Lord has given me*.*”* ([42], p.937). Religion and spiritual beliefs shape their philosophy of life and can also relieve thoughts about death “*I am not afraid of dying […] I have had a good life and I look confidently forward to an even better after life*.*”* ([39], p.205). Some older adults rely on their God and turn to him to help overcome problems. Faith gives them comfort, peace of mind and a sense of meaning and purpose in life, and makes them look forward to each day. Volunteering and taking part in **religious activities** and practices, such as going to church, were described as ways to stay socially active and involved.

However, there was large variation in the value older adults attributed to religion and faith. Some respondents commented that religion was not important at all: “*Well I haven’t any faith in a personal God that’s looking after you*. *If you know anything about astronomy you realise how stupid that is*.” ([57], p.1895).

**Spirituality** is often mentioned as synonymous with religion. Yet, it may also encompass non-religious beliefs and rituals, such as meditation, self-development and turning attention to the inner life: “*I’m spiritually—not religious–I’m spiritually very calm with myself*. *I discovered things about myself in mid to later years that have been sitting there all the time*.” ([75], p.570). For some older adults ageing means a certain kind of spiritual growth and becoming a wiser or better person.

#### Home and neighbourhood: Feeling secure at home and living in a pleasant and accessible neighbourhood

The **meaning of home** for older adults often extends that of ‘just a residence’; it brings about feelings of staying in a pleasant place with familiar and important objects and shared norms, history and values [49]. This is illustrated by a respondent: “*It’s also important to have a safe place to live*. *It means a lot to have a flat that is adapted to your needs*.*”* ([36], p.46). A home provides shelter, a place where one can feel safe and comfortable, and it is a private domain in which you are in control and can maintain your daily routines: “*my home is my castle*” ([39], p.212).

Many older adults strive to keep **living at home** or in the community for as long as possible and preferred adapting the home environment rather than moving; “*Well*, *the first condition is to stay fit enough to be able to live on your own*. *And to live at home; I'd much rather live here at home than in some institution*” ([72], p.54).

When becoming dependent on other people or services, the feeling of **safety** and privacy at home can be at stake. Older adults addressed how home adaptations and alarm systems increase their feelings of safety. Some were concerned about safety from crime in and around their home: “*After dark*, *we never go out*. *And we never answer the door unless we’re expecting somebody; otherwise*, *our door remains closed*” ([74], p.271).

Having friendly neighbours and experiencing a sense of familiarity in your **neighbourhood** can enable a sense of security: “*I suppose it’s security*, *ain’t it—you’ve been in the area for a long time*, *you know where everything is*, *you know everybody locally—you feel more secure*” ([57], p.1894). Neighbours and social networks can provide help in everyday life and social contact. Furthermore, according to older adults QoL is enhanced by a pleasant environment which provides the opportunity to enjoy nature and do interesting activities.

However, some environmental barriers were mentioned that restricts older people from going out. These are for example poor weather and obstacles such as ‘poorly maintained sidewalks, and street/traffic designs poorly adapted to older adults’ [76]. **Accessibility** of the environment is a key to participation in community life [70]. According to Bowling et al. [40], lack of easy access to reliable, cheap and convenient public transport was mentioned as inhibiting social contacts and activities. Access to community services such as post offices and libraries was mentioned to be important as well.

#### Financial security: Not feeling restricted by your financial situation

Good financial circumstances make life easier. Respondents mentioned their ability to meet basic needs, for example: “*It’s having* ***sufficient money****… to do what you require*, *run your car*, *say*, *and pay your bills*, *and have the odd holiday… sufficient money not to have to worry about money*” ([41], p.838). Income affects people’s capacity to pay for services such as home-help, physiotherapy and meals, or transport. It seemed that especially respondents who experienced financial difficulties mentioned finances as important for QoL.

Adequate finances are not only important to afford basic essentials and household bills, they also provide **freedom**: the possibility to afford outings, activities and extras. Older people can feel restricted by a lack of financial resources which hinders them to enjoy themselves and to participate in society (e.g. through holidays, going to the cinema, inviting friends for dinner, a train ride, luxuries) and to feel independent and secure (living comfortably and without worries). Respondents lacking sufficient money felt unable to enjoy life. “*There are so many things I would like to do that I can’t afford and that is not fun*” ([39], p.212).

Next to having sufficient money, respondents mention how **material and (housing) conditions** help to feel comfortable and retain independence: “*I should buy myself one of those adjustable beds*. *And a chair that I could stand up (from) easily […] Well*, *I’m talking millionaire*, *now*, *I would like a bath that you could get into easier*, *and all things that … I could be independent until … the rest of my days*.” ([55], p.687).

### Quality of life as a web of intertwined domains

The domains should not be interpreted as clearly demarcated and distinct units. Our findings rather imply that QoL can be understood as a dynamic web of domains, where each domain covers a swarm (or cluster) of strongly connected QoL aspects. During the analysis it became clear that the subthemes that describe the QoL domains sometimes overlap and are strongly connected with each other. Within quotations, these connections were visible by statements about how QoL aspects affect each other, for example: “*If you have a good mental outlook*, *you are probably going to be active and you're probably going to eat reasonably well*” ([63], p.s34). Several connections and points of overlap between QoL aspects are exposed in the domain descriptions above, for example, the idea that being able to mean something for other people is important for your own QoL is part of the subtheme ‘helping others’ (domain Role and Activity) but also of the subtheme ‘reciprocality’ (domain Relationships). Moreover, it is strongly connected to the subthemes ‘being a burden’ (domain Autonomy) and ‘self-worth’ (domain Role and activity).

The domains are thus intertwined; they interact with each other and sometimes even partly fuse. However, a clear hierarchical picture of QoL domains, in which some domains can be identified as purely instrumental to other domains, was not found. Health was for example said to be needed for QoL aspects covered in other domains such as ‘Role and activity’ and ‘Relationships’, but certain activities and relationships also contribute to one’s ‘Health perception’.

All the interactive threads between the QoL aspects make the web dynamic: if something occurs in one of the domains it influences the rest of the web. This implies that the status of a domain for an individual is affected by the status of the other domains at that time.

### Coverage of the QoL domains

Table 3 shows the ‘coverage’ of the domains in the individual studies. As such, it provides an overview of how extensive and consistently the QoL aspects that we categorised into these domains were mentioned across settings and subgroups of older adults. For example, the domains ‘Autonomy’, ‘Role and occupation’, ‘Attitude and adaption’, ‘Health perception’ and ‘Relationships’ were covered in almost all studies, suggesting that the QoL aspects categorised into theses domains are important across settings and groups. These QoL aspects were most detailed and extensively described in the studies and are therefore more prominent in our synthesis. QoL aspects categorised into the domains ‘Spirituality’ and ‘Financial security’ were less universal as they were less consistently covered across studies.

**Table 3:** Coverage of core domains by the included papers.

Domain	Number of studies	Paper references																																															
		[35]	[33]	[36]	[37]	[38]	[39]	[40]	[41]	[42]	[43]	[44]	[45]	[46]	[47]	[48]	[49]	[50]	[51]	[32]	[52]	[53]	[54]	[55]	[56]	[57]	[58]	[59]	[60]	[61]	[62]	[63]	[64]	[65]	[66]	[67]	[68]	[69]	[70]	[71]	[72]	[73]	[74]	[75]	[76]	[77]	[31]	[34]	[78]
Health perception	41/48	**	-	**	*	**	**	***	**	***	*	***	**	**	**	**	**	*	**	*	-	**	-	**	**	**	-	***	**	**	**	***	*	**	*	**	**	-	**	*	**	*	**	-	*	*	**	-	**
Autonomy	39/48	***	***	*	-	***	*	***	***	***	*	-	-	**	-	*	**	*	*	**	*	***	***	***	-	**	*	*	**	*	***	**	**	**	-	-	***	*	*	*	***	*	*	-	**	***	-	*	***
Role and activity	41/48	***	*	***	***	***	**	***	**	***	-	***	*	***	**	***	***	-	-	***	-	*	**	***	***	***	-	-	**	***	**	***	**	**	**	***	**	***	***	*	**	*	***	***	**	-	**	**	***
Relationships	40/48	*	**	***	***	*	***	***	**	***	*	**	**	*	**	***	**	*	**	*	*	**	-	***	-	***	*	**	**	***	**	-	***	**	-	-	*	**	**	*	***	*	**	-	*	*	-	*	-
Attitude and adaptation	39/48	***	*	***	-	-	***	**	**	***	-	***	**	**	-	***	**	-	**	***	*	***	-	***	**		*	***	***	***	***	***	***	**	*	**	**	**	***	-	**	**	**	**	*	***	**	*	-
Emotional comfort	35/48	**	-	*	**	-	***	***	-	**	-	-		*	-	*	*	-	***	*	*	***	**	***	-	*	**	-	-	***	**	**	***	*	**	-	*	**	**	*	**	*	**	*	*	-	**	*	***
Spirituality	22/48	-	-	-	*	*	**	*	-	*	-	-	**	-	-	**	-	-	*	-	-	-	-	-	-	**	-	-	*	**	**	**	*	-	-	-	-	**	-	-	**	*	**	**	**	**	*	-	-
Home and neighbourhood	31/48	*	**	**	*	***	**	***	**	-	*	-	-	-	**	-	***	*	*	-	-	*	*	***	**	**	*	**	-	**	**	-	**	-	*	-	**	-	***	-	**	-	***	-	**	-	**	-	**
Financial security	25/48	-	-	-	**	*	**	***	**	*	**	*	-	-	-	**	-	-	-	**	-	*	*	***	-	**	-	-	**	**	**	-	**	**	*	-	-	-	**	*	-	-	**	-	**	-	*	-	-

- not mentioned

* Briefly mentioned

** Discussed

*** Discussed extensively

## Discussion

### Main findings

The purpose of this review was to synthesize the findings of qualitative studies that explored what QoL means to older adults living at home. The synthesis of 48 studies resulted in a categorization of QoL aspects into nine QoL domains: ‘feeling healthy and not limited by your physical condition’ [Health perception], ‘being able to manage on your own, retaining dignity and not feeling like a burden’ [Autonomy], ‘spending time doing activities that bring a sense of value, joy and involvement’ [Role and activity], ‘having close relationships which makes you feel supported and enable you to mean something for others’ [Relationships], ‘looking on the bright side of life’ [Attitude and adaptation], ‘feeling at peace’ [Emotional comfort], ‘feeling attached to and experiencing faith and self-development from beliefs, rituals and inner reflection’ [Spirituality], ‘feeling secure at home and living in a pleasant and accessible neighbourhood’ [Home and neighbourhood], and ‘not feeling restricted by your financial situation’ [Financial security].

While we distinguished these nine domains, the quotes from older adults often fitted into more than one domain and the quotes exposed numerous connections between domains. This implies that the concept of QoL can be understood as a dynamic web of domains meaning that the domains are strongly intertwined and are affected by the status of the other domains at that time.

### Reflection on findings

Although it is often claimed by researchers [53,73] that little research has been done into the perspective of older adults on QoL to motivate the relevance of their empirical study, this review proves the opposite. There is a wealth of material available covering more than 25 years of research.

All authors of the primary studies used some sort of sub-classification of the themes they identified, which confirms that QoL is perceived as a multidimensional concept. The various ways in which primary authors labelled, classified and made sense of QoL aspects mentioned by older adults can be explained by our understanding of QoL as a dynamic web of domains that strongly interact and partly overlap. However, we found that the content of the quotes from older adults themselves was very similar across the studies, showing common experiences and use of language to describe these experiences and the contribution to QoL. This means that under these diverse interpretations and conclusions there is something more universal in the meaning of QoL for older adults living at home in Western societies. Rather than using the more theoretical interpretations of QoL by the primary authors for our classification system we focused primarily on the quotes of older adults in order to grasp these universal experiences. Furthermore, our synthesis is a more comprehensive classification of QoL aspect compared to the QoL classifications in the primary studies.

In some of the included papers, domains such as ‘Health perception’, ‘Home and neighbourhood’, ‘Spirituality’ and ‘Financial security’ were characterized as instrumental to or as a resource for other QoL domains, because some older adults mention these themes merely as helping them to adjust and do the things they would like to do. In a similar vein, themes described under ‘Attitude and adaptation’ for example are often about strategies to improve QoL or maintain a good QoL. However, there are also fragments showing that these domains are important in itself or are influenced by aspects covered in the other domains, such as the activities that one participates in. It is therefore difficult to draw a line between domains that are more instrumental to QoL and domains that should be seen as ultimate QoL components, i.e. in cause and effect relation.

The domains ‘Health perception’, ‘Autonomy’, ‘Role and activity’, ‘Attitude and adaption’ and ‘Relationships’ were most extensively and universally covered across settings and participants. Although this suggest that these domains are most distinctive in characterising QoL, we think that the coverage of the domains not necessarily reflects the importance of these domains for QoL as conceived by older adults. For example, the domains ‘Spirituality’ and ‘Financial security’ were less extensively and consistently covered, but they seem very important for specific groups or individuals. Our list of domains should therefore be seen as a comprehensive framework of potentially important QoL aspects. The relative importance and meaning of the QoL domains depend on one’s circumstances but also on personal preferences. This can be taken into account in a QoL measure by giving respondents the opportunity to indicate how their current situation relates to their needs in/wishes for certain domains as is for example done in the ASCOT [79,80].

The synthesis affirms that adaptation processes can result in a shift in perceived importance of domains, as well as in an altered understanding of what these domains mean to someone, also known as ‘response shift’ [79, 80]. For example, in case of declining health and independence, older adults seem to focus their description of health and independence on their remaining abilities. Rather than a strict or technical definition in terms of physical functioning (which may result in a characterisation of themselves as ‘unhealthy’ or ‘dependent’ persons), they describe health in comparison with others of their age and in terms of ability to adapt and to carry out meaningful activities (which, in turn may shift from more difficult goals such as gardening to more easy tasks such as grooming and eating). So, both the importance an individual places on and understanding an individual has of each of the QoL domains are dynamic and change over the course of life.

### Comparison with other studies

Stanley and Cheeck [18] only identified and reviewed four qualitative studies that investigated older adults’ perspective on well-being, and the results of these studies were not synthesized whereas we synthesized findings of 47 studies. The activities and strategies listed in their review, however, are comparable to the domains identified in our synthesis.

In an earlier review by Brown et al. [19]), it was stated that QoL can theoretically encompass a wide ranging array of domains. The QoL components Brown et al identified from a mix of 45 surveys and qualitative studies among older adults were remarkably consistent to the themes identified in our review. Some of our subthemes like ‘Home and neighbourhood’ (a good home, safety, transport facilities and a pleasant environment) were mentioned in the review by Brown et al., but were allocated to other domains. Nothing was mentioned in the review by Brown et al. about ‘Attitude and adaptation’, while we found that older adults consider the ability to adapt to difficult circumstances essential for a good QoL.

### Strengths and limitations

This study contributes to our understanding of QoL from the perception of older adults as it brings together the findings of 48 studies, covering views of more than 3,400 community-dwelling older adults from 11 Western countries. The synthesis therefore provides a more universal understanding of QoL than each study on its own. By recoding and synthesising the reported data rather than summing up a list of identified themes we aimed for a ‘whole’ greater than the sum of its parts.

The samples from the included studies were selected from very diverse populations, covering younger and older age groups, and active volunteers as well as vulnerable older adults. Although, we recognize that diversity of respondents/samples may influence how they characterize quality of life, our aim was to define the commonalities over these diverse respondents. Therefore, we analyzed the content of respondents’ quotations, not their personal characteristics. Considering the fact that diverse populations were included, we are confident that our findings cover a wide range of views on what QoL means to older adults. Although some QoL domains prevailed more often in specific contexts, the identified domains were fairly consistent across the studies. This contributes to the credibility of the findings.

In our study and in the selection of articles, we did not use a fixed definition of QoL, but rather we employed a broader view on QoL. This is among others shown by our broad search which included search terms such as ‘life satisfaction’, ‘successful aging’ ‘living well’, ‘well-being’. Our goal was to include all studies describing the perspective of older adults on quality of life, and not to engage in the theoretical discussion about existing definitions. An advantage of using such a broad approach is that the QoL domains presented here are all based on what is important to respondents themselves. A disadvantage might be that it is not clear how the domains identified in our study relate to other concepts of QoL, well-being or successful aging. Our broad search strategy is reflected in the fact that we only identified one additional paper from the reference list of one of the included studies. Thus, we are confident that we included virtually all relevant studies.

Because we found that similar QoL aspects were identified across the studies and because our coding scheme did not change much after analysing the first half of papers, we think that our QoL framework does not need to be revised considerably when new qualitative studies on this topic will be added. There may however be themes that older adults or interviewers are reluctant to address. One subtheme for example that is surrounded by an ‘audible silence’ [24] is intimacy and sexuality in old age. Although large parts of the texts in the papers concern relationships, hardly anything was reported about the importance of experiencing intimacy. It might be that this is a theme not considered important by older adults, but it also possible that they are reluctant to share their thoughts about this topic in interviews that focus on QoL [81].

To enhance the transparency about the origin and background of the QoL aspects identified in our synthesis we provided a table showing which QoL domains were covered in which papers (Table 3) and a table showing the ‘signature features’ [21] of the included studies, such as the sampling strategy, the focus of the study and the methodological orientation and theoretical frameworks used. Individual interviews as well as focus groups were included. The variety in signature features supports the conclusion that are findings are not influenced by one specific methodological orientation or theoretical framework.

However, there are limitations to condensing such a wealth of material. Sandelowski et al.’s question ‘Can you sum up a poem?’ ([21], p.366) very well reflects the challenge of synthesising qualitative studies. A synthesis undeniably leads to a selection of material which in itself already is the result of a process of condensing and interpretation of primary raw data done by the authors of the studies. We therefore refer readers to the individual papers if they are interested in more detailed discussions and interpretations of the identified domains. Tables 2 and 3 can serve as a guide for identifying relevant studies. Opponents of synthesising qualitative studies remark that the loss of explanatory context is a cause for concern as qualitative findings are often specific to a particular context, time and group of participants [21, 23]. We attempted to preserve some context by providing summaries and signature features of each study in Table 2.

A second caution concerns our own perspective and professional and personal backgrounds. Our classification of QoL domains as resulting from the synthesis is influenced by our own values, background and experiences, just like the various classification systems as constructed by the primary authors of the included studies. Our research is mainly health-oriented and less socially oriented, which likely shaped our interpretation of the experience of QoL by older adults living at home. Yet, the possible effect of our personal backgrounds is reduced by the variation of the authors and the thoroughness of the peer review process in all phases of the study.

Finally, we did not perform a formal quality appraisal of the included studies. Researchers vary in their opinion on the use of quality appraisal of qualitative studies in a synthesis [20, 21, 23, 24, 26, 28, 82–84]. Apart from the diverse opinions concerning what a ‘good’ qualitative study is, there is no consensus about the most appropriate role of such an appraisal. For example, Atkins et al. [23] experienced that appraising studies became an exercise in judging the quality of the written report rather than the research procedure itself. Campbell et al. [24], therefore, argued that only studies with fatal flaws should be excluded. Based on a subjective overall evaluation going from our personal experience with doing and reporting qualitative studies, we did not consider any one of the included studies as fatally flawed so did not see a reason to exclude one of them from the synthesis. Furthermore, because papers with mainly short descriptions and explanations of the data offer in general few insights, in several studies it was found that poorer quality qualitative studies contribute only minimally to a synthesis, adding weight to the better studies [23, 24, 26]. We found in our study as well that the studies that provided the most conceptually rich descriptions and comprehensive quotations of QoL aspects (as indicated in Table 3) automatically contributed most to our synthesis. Finally, assessment of quality is much more important when themes of the included studies are used. Since we did not use the codes used by researchers of the included studies, but defined a set of themes and codes ourselves that we applied to the data extracted from the included studies, we considered it unnecessary to assess the quality of the included studies.

### Implications for care practice, measurement of QoL and further research

Stanley and Cheek’s [18] conclusion that the older person’s subjective perspective on well-being is lacking in the literature, does not hold anymore given the fair amount of studies that we have identified. Our review provides a guide to researchers by providing an extensive overview of individual qualitative studies providing a complete overview of QoL domains and subthemes and references to more specific sources.

Our findings show that autonomy and living at home are valued by older adults, which is in line with current ageing in place policies. Because the QoL web is dynamic and consists of intertwined domains, such policies likely affect other QoL domains and may result in negative consequences as well, for example in loneliness and safety. With more and more older adults ageing at home there will be an inevitable increased need for care services and support. Care and support for older adults living at home should be aimed at improving or maintaining QoL, foremost on the QoL domains that they value themselves. Perhaps the most important practical implication of this review is that a broad definition of QoL should be targeted, encompassing various interrelated domains. The identified domains can provide direction to care services and support, and consequences on all domains should be considered when developing personal care plans. These domains can be used in personal conversations about how older adults’ QoL could be improved and to personalize their care. Because the QoL web is dynamic such plans should be updated and discussed regularly. Furthermore, although it can be discussed to what extent care services and support are responsible for all identified QoL domains in this synthesis, the domains should be considered as outcomes or important side effects in improvement processes, evaluation, monitoring or benchmarking of care services and support for older adults living at home.

The findings of our study may also be used by researchers developing new QoL measures for older adults. Currently, not all of the identified domains are sufficiently covered in existing QoL measurement instruments for older adults. Many available instruments focus on *health-*related QoL and include physical and psychological dimensions [15, 16, 85, 86], especially the preference-based measures developed for use in economic evaluations [14, 17]. And preference-based measures adopting a broader perspective on QoL do not include a health dimension [87, 88]. Although these measures may be suitable for specific purposes, our findings suggest that from the point of view of older adults important domains are missing. Further research on the operationalisation and measurement of QoL domains least frequently covered (Autonomy, Role and activity, Attitude and adaptation, Spirituality, Home and neighbourhood, and Financial security) is recommended in order to increase the face validity of QoL instruments for older adults. Development of measurement instruments based on a broad definition of QoL in line with older adults’ perspective may help guide care services to direct their policies at what is important for older adults.

Nonetheless, several findings from this review indicate that incorporating older adults’ perspective in the measurement of QoL can be challenging. Especially the dynamic character of QoL and shifting reference points and concept definitions over time by older adults, suggest that it may be problematic to use generic, static instruments. Adaptive, flexible ways of measurement are probably more in line with the characterisation of QoL found in this review. We also recommend to carefully align the choice or development of instrument to the goals and setting of measurement.

Furthermore, the interrelatedness of domains and absence of strict boundaries between domains mean that for older adults, any classification of QoL domains may feel artificial. As Hendry & McVittie [58] put it: “*people do not segment their lives into component parts*”.

### Conclusions

We identified nine QoL domains from 48 qualitative studies among different groups of older adults. Older adults value feeling healthy and not limited by their physical condition, being able to manage on their own, retaining dignity and not feeling like a burden, spending time doing activities that bring a sense of value, joy and involvement, having close relationships which makes them feel supported and enable them to mean something for others, looking on the bright side of life, feeling at peace, feeling attached to and experiencing faith and self-development from beliefs, rituals and inner reflection, feeling secure at home and living in a pleasant and accessible neighbourhood and not feeling restricted by their financial situation. Which domains apply in a specific situation needs to be decided by service providers and care professionals themselves, preferably by turning on specific domains using flexible measurement instruments. However, it is important that service providers and care professionals realize that the QoL domains are strongly intertwined meaning that changes in one domain likely affect other QoL domains.

## Supporting information

S1 File Search strategies per database. (DOCX)

S2 File PRISMA checklist_Thematic synthesis QoL. (DOCX)
