# Supplementary material for: What does quality of life mean to older adults? A thematic synthesis
Source: PLoS One. 2019 Mar 8;14(3):e0213263. doi: 10.1371/journal.pone.0213263 (PMC6407786; doi:10.1371/journal.pone.0213263)
Supplement: S1 File — (DOCX) [file pone.0213263.s001.docx]

**S1 File. Search strategies per database**

**Search strategy for PubMed (28 November 2018)**

[Mesh] = Medical subject headings

[tiab] = words in title OR abstract

| **Search** | **Query** | **Items found** |
| --- | --- | --- |
| **#5** | (#1 AND #2 AND #3 AND #4) | **5730** |
| **#4** | (("Qualitative Research"[Mesh] OR "Focus Groups"[Mesh] OR qualitative research*[tiab] OR qualitative method*[tiab] OR qualitative approach*[tiab] OR focus group*[tiab] OR (("Interview" [Publication Type] OR "Interviews as Topic"[Mesh] OR interview*[tiab]) AND (“semi-structured”[tiab] OR semistructured[tiab] OR unstructured[tiab] OR structured[tiab] OR informal*[tiab] OR “in-depth”[tiab] OR indepth[tiab] OR guide[tiab] OR guides[tiab] OR qualitative[tiab])))) | **174848** |
| **#3** | ("Narration"[Mesh] OR perspective*[tiab] OR opinion*[tiab] OR perception*[tiab] OR experienc*[tiab] OR view[tiab] OR views[tiab] OR definition*[tiab] OR attribute*[tiab] OR component*[tiab] OR domain*[tiab] OR contribut*[tiab] OR importan*[tiab] OR meaning*[tiab] OR understand*[tiab] OR concept*[tiab] OR narrati*[tiab]) | **6928738** |
| **#2** | ("Quality of Life"[Mesh] OR "Happiness"[Mesh] OR "Personal Satisfaction"[Mesh] OR life qualit*[tiab] OR "quality of life"[tiab] OR qol[tiab] OR happiness*[tiab] OR life satisf*[tiab] OR well being[tiab] OR wellbeing[tiab] OR ageing well[tiab] OR aging well[tiab] OR successful aging[tiab] OR successful ageing[tiab]) | **367007** |
| **#1** | ("Aged"[Mesh] OR elder*[tiab] OR older adult*[tiab] OR older people*[tiab] OR senior[tiab] OR seniors[tiab] OR oldest old[tiab] OR nonagenari*[tiab] OR octogenari*[tiab] OR centenari*[tiab] OR older person*[tiab]) | **2990234** |

**Search strategy for Ebsco/PsycInfo (28 November 2018)**

DE = descriptors, keywords

TI = words in title

AB = words in abstract

| **#** | **Query** | **Limiters/Expanders** | **Results** |
| --- | --- | --- | --- |
| **S19** | S10 AND S12 AND S13 AND S18 |  | **5,579** |
| **S18** | S5 OR S7 OR S15 OR S17 |  | **294,118** |
| **S17** | S9 AND S16 |  | **151,648** |
| **S16** | S6 OR S8 OR S14 |  | **428,053** |
| **S15** |  | Limiters - Methodology: -Focus Group, QUALITATIVE STUDY | **207,829** |
| **S14** |  | Limiters - Methodology: INTERVIEW | **239,619** |
| **S13** | S3 OR S4 |  | **177,153** |
| **S12** | S1 OR S2 OR S11 |  | **546,758** |
| **S11** | DE "Aging" OR DE "Aging in Place" OR DE "Physiological Aging" |  | **74,034** |
| **S10** | TI (perspective* OR opinion* OR perception* OR experienc* OR view OR views OR definition* OR attribute* OR component* OR domain* OR contribut* OR importan* OR meaning* OR understand* OR concept* OR narrati*) OR AB (perspective* OR opinion* OR perception* OR experienc* OR view OR views OR definition* OR attribute* OR component* OR domain* OR contribut* OR importan* OR meaning* OR understand* OR concept* OR narrati*) |  | **2,342,030** |
| **S9** | TI (“semi-structured” OR semistructured OR unstructured OR structured OR informal* OR “in-depth” OR indepth OR guide OR guides OR qualitative) OR AB (“semi-structured” OR semistructured OR unstructured OR structured OR informal* OR “in-depth” OR indepth OR guide OR guides OR qualitative) |  | **351,612** |
| **S8** | TI (interview*) OR AB (interview*) |  | **299,426** |
| **S7** | TI (“qualitative research*” OR “qualitative method*” OR “qualitative approach*” OR “focus group*”) OR AB (“qualitative research*” OR “qualitative method*” OR “qualitative approach*” OR “focus group*”) |  | **58,819** |
| **S6** | DE "Interviews" |  | **8,164** |
| **S5** | DE "Qualitative Research" |  | **20,937** |
| **S4** | TI (“life qualit*” OR "quality of life" OR qol OR happiness* OR “life satisf*” OR “well being” OR wellbeing OR “ageing well” OR “aging well” OR “successful aging” OR “successful ageing”) OR AB (“life qualit*” OR "quality of life" OR qol OR happiness* OR “life satisf*” OR “well being” OR wellbeing OR “ageing well” OR “aging well” OR “successful aging” OR “successful ageing”) |  | **151,139** |
| **S3** | ((DE "Quality of Life") OR (DE "Happiness")) OR (DE "Satisfaction" OR DE "Life Satisfaction" OR DE "Marital Satisfaction" OR DE "Need Satisfaction" OR DE "Role Satisfaction") |  | **80,066** |
| **S2** | TI (aged OR elder* OR “older adult*” OR “older people*” OR senior OR seniors OR “oldest old” OR nonagenari* OR octogenari* OR centenari* OR “older person*”) OR AB (aged OR elder* OR “older adult*” OR “older people*” OR senior OR seniors OR “oldest old” OR nonagenari* OR octogenari* OR centenari* OR “older person*”) |  | **350,991** |
| **S1** |  | Limiters - Age Groups: Aged (65 yrs & older) | **299,333** |

**Search strategy for Ebsco/CINAHL (28 November 2018)**

MH = mapped heading, keywords

+ = mapped headings with explosion

TI = words in title

AB = words in abstract

| **#** | **Query** | **Limiters/Expanders** | **Results** |
| --- | --- | --- | --- |
| **S11** | S3 AND S4 AND S9 AND S10 |  | **4,449** |
| **S10** | S5 OR S8 |  | **185,644** |
| **S9** | TI (perspective* OR opinion* OR perception* OR experienc* OR view OR views OR definition* OR attribute* OR component* OR domain* OR contribut* OR importan* OR meaning* OR understand* OR concept* OR narrati*) OR AB (perspective* OR opinion* OR perception* OR experienc* OR view OR views OR definition* OR attribute* OR component* OR domain* OR contribut* OR importan* OR meaning* OR understand* OR concept* OR narrati*) |  | **1,231,815** |
| **S8** | S6 AND S7 |  | **91,629** |
| **S7** | TI (“semi-structured” OR semistructured OR unstructured OR structured OR informal* OR “in-depth” OR indepth OR guide OR guides OR qualitative) OR AB (“semi-structured” OR semistructured OR unstructured OR structured OR informal* OR “in-depth” OR indepth OR guide OR guides OR qualitative) |  | **227,989** |
| **S6** | (MH "Interviews+") OR ( TI (interview*) OR AB (interview*) ) |  | **258,198** |
| **S5** | ( (MH "Qualitative Studies+") OR (MH "Focus Groups") ) OR ( TI (“qualitative research*” OR “qualitative method*” OR “qualitative approach*” OR “focus group*”) OR AB (“qualitative research*” OR “qualitative method*” OR “qualitative approach*” OR “focus group*”) ) |  | **150,414** |
| **S4** | ( (MH "Quality of Life+") OR (MH "Happiness") OR (MH "Personal Satisfaction") ) OR ( TI (“life qualit*” OR "quality of life" OR qol OR happiness* OR “life satisf*” OR “well being” OR wellbeing OR “ageing well” OR “aging well” OR “successful aging” OR “successful ageing”) OR AB (“life qualit*” OR "quality of life" OR qol OR happiness* OR “life satisf*” OR “well being” OR wellbeing OR “ageing well” OR “aging well” OR “successful aging” OR “successful ageing”) ) |  | **183,367** |
| **S3** | S1 OR S2 |  | **689,472** |
| **S2** | ( (MH "Aged+") OR (MH "Aging+") OR (MM "Attitude to Aging") ) OR ( TI (aged OR elder* OR “older adult*” OR “older people*” OR senior OR seniors OR “oldest old” OR nonagenari* OR octogenari* OR centenari* OR “older person*”) OR AB (aged OR elder* OR “older adult*” OR “older people*” OR senior OR seniors OR “oldest old” OR nonagenari* OR octogenari* OR centenari* OR “older person*”) ) | Limiters - Age Groups: Aged: 65+ years, Aged, 80 and over | **689,472** |
| **S1** |  | Limiters - Age Groups: Aged: 65+ years, Aged, 80 and over | **689,472** |

Bottom of Form
